# Expression of TNF-*α*, April and BCMA in Behcet's Disease

**DOI:** 10.1155/2014/380405

**Published:** 2014-11-05

**Authors:** Olfat G. Shaker, Shereen O. Tawfic, Amira M. El-Tawdy, Mohamed H. M. El-Komy, Manal El Menyawi, Ahmed A. Heikal

**Affiliations:** ^1^Department of Medical Biochemistry and Molecular Biology, Faculty of Medicine, Cairo University, Giza, Egypt; ^2^Department of Dermatology, Faculty of Medicine, Cairo University, Giza, Egypt; ^3^Department of Internal Medicine, Kasr Al-Aini Hospital, Faculty of Medicine, Cairo University, Giza, Egypt

## Abstract

*Background*. Tumor necrosis factor-alpha (TNF-*α*) is an important proinflammatory cytokine which plays an important role in the immunopathogenesis of Behcet's disease (BD). B cell activating factor (BAFF) and its homolog A proliferation inducing ligand (APRIL) are members of the tumor necrosis factor family. BAFF binds to 3 receptors, B cell activating factor receptor (BAFF-R), transmembrane activator and calcium modulator ligand interactor (TACI), and B cell maturation antigen (BCMA) that are expressed by B cells. *Objective*. Estimation of the serum levels of TNF-*α*, APRIL, BAFF, and BCMA in patients with BD in an effort to evaluate their degree of involvement in the pathogenesis and development of BD. *Patients and Methods*. This study included 30 male patients fulfilling the international study group criteria for the diagnosis of BD. Twenty age-matched healthy male volunteers served as control. Serum samples were used for quantification of TNF-*α*, APRIL, BCMA, BAFF, and hsCRP using ELISA techniques. *Results*. The mean serum levels of TNF-*α*, APRIL, BCMA, and BAFF were more elevated in cases than in controls in a statistically significant manner (*P* < 0.001). Positive correlation was observed between hs-CRP and BDCAF (Behcet's disease current activity forum) index (*r* 0.68, *P* < 0.001). None of the TNF family members tested was affected by a positive pathergy test. *Conclusions*. Patients have significantly higher levels of TNF family members' (TNF-*α*, BAFF, APRIL, and BCMA) compared to controls which might contribute to the pathogenesis of BD.

## 1. Introduction

Behcet's disease (BD) is a multisystem inflammatory disorder, currently classified as a vasculopathy. Its etiopathogenesis is unclear, but environmental, genetic, and autoimmune factors have been considered. There have been ongoing efforts to elucidate the aetiology of BD [1].

Tumor necrosis factor-alpha (TNF-*α*) is an important proinflammatory cytokine which plays an important role in the immunopathogenesis of Behcet's disease. The serum level of TNF-*α* is elevated in patients with BD, and a dramatic response to anti-TNF-alpha antibody treatment further supports the role of TNF in BD [2]. It has been demonstrated that neutrophils from patients with Behcet's disease constitutively express TNF-mRNA and produce increased amounts of TNF with lipopolysaccharide stimulation, which may play a key role in the generation of Th1 immune responses. Enhanced TNF production might help to autoprime neutrophils and prolong their own life-span, which might result in accumulation of activated neutrophils in the site of inflammation [3]. Moreover, soluble tumour necrosis factor receptors sTNFR1 and sTNFR2 are produced at sites of inflammation and are markers of arthritis activity in Behcet's disease [4].

B cell activating factor (BAFF) and its homolog A proliferation inducing ligand (APRIL) are members of the tumor necrosis factor family and are expressed by monocytes, dendritic cells (DC)s, neutrophils, basophils, stromal cells, activated T cells, activated and malignant B cells, and epithelial cells [5]. BAFF binds to 3 receptors, B cell activating factor receptor (BAFF-R), transmembrane activator and calcium modulator ligand interactor (TACI), and B cell maturation antigen (BCMA), that are expressed by B cells at various times during their ontogeny [6]. BAFF-R is specific for BAFF whereas TACI and BCMA also bind to APRIL [7]. BAFF was found upregulated in the peripheral circulation and in skin biopsies from BD patients [1]. To our knowledge, the expression of APRIL and BCMA in Behcet's disease has not been examined before. Thus we aimed in this work to evaluate the serum levels of TNF-*α*, APRIL, BAFF, and BCMA in patients with BD in an effort to evaluate their role in this disease and their relation to the disease activity.

## 2. Patients and Methods

This study included thirty Egyptian male patients with BD fulfilling the international study group criteria for the diagnosis of BD [8]. Patients with other autoimmune diseases or inflammatory disorders unrelated to BD were not included. All patients were recruited from Rheumatology, Dermatology Outpatient Clinics, and Internal Medicine Inpatient Department of Cairo University Hospitals (Kasr El-Aini hospital).

Patients with other associated disorders unrelated to BD including hepatic or renal diseases, diabetes, or essential hypertension were not included in this study. The nature of the study was explained to all participants. The study was approved by the Ethical Committee of Faculty of Medicine, Cairo University, and an oral and written consent was obtained from all participants. The ethical committees approve all the consent procedures.

### 2.1. Patient Group

We selected 30 patients fulfilling ISG criteria for the diagnosis of BD from outpatient clinic of rheumatology and dermatology and those admitted to internal medicine departments. We excluded patients with any disorder as hepatic, renal disease, diabetes, other autoimmune diseases, or essential hypertension. The patients were grouped as active or inactive at the time of the study, and the patients who met at least one criterion of the ISG for BD were considered to be in the active stage of the disease. Patients who had been free of lesions for the previous 30 days or more were accepted as having inactive BD.

Twenty patients received immunosuppressives and the other 10 patients were controlled on colchicines. So, at the time of sampling 20 patients were active and 10 were in remission.

Patients were subjected to the following: complete medical assessment including medical history and complete physical examination with special emphasis on Behcet's disease symptoms and signs including vascular involvement either arterial or venous. Fundus examination assessment was done by a slit lamp examination for detection of any ocular manifestations reported in Behcet's disease, for example, anterior uveitis, posterior uveitis, iridocyclitis, keratitis, retinal vasculitis, retinal vein occlusion, and optic neuritis [9]. Assessment of disease activity using Behcet's disease current activity form (BDCAF) was performed to all our patients according to the method presented by Bhakta et al., in 1999 [10]. Pathergy test of all patients was performed by intradermal puncture under the forearm skin with 20-gauge needle, 5 mm deep obliquely under sterile conditions and without injecting saline. Pathergy test is considered positive if a sterile pustule of 3–10 mm was observed after 24–48 h at the needle prick site in the skin [11]. Imaging studies: duplex and colored coated Doppler examination on arterial and venous systems were done when clinically indicated and the symptoms of the patients required this maneuver to detect any arterial or venous occlusions.

### 2.2. Control Group

Twenty age-matched healthy male volunteers were recruited and served as a control group. Control subjects with history or signs of recent acute infection or chronic disease (e.g., other autoimmune or atopic disorders and no previous history of recurrent aphthous) were not included.

Control subjects are age-matched and sex-matched healthy male volunteers from similar ethnic background and we excluded those with recent acute infection.

### 2.3. Samples

Peripheral blood was obtained by venipuncture from patients and healthy adult controls using an Institutional Review Board approved protocol and informed consent. Serum was separated to be used for measurements of TNF-*α*, BAFF, BCMA, APRIL, and hsCRP.

Serum BAFF levels were measured using a specific ELISA kit (R&D Systems, Minneapolis, MN) according to the manufacturer's protocol. This assay employs the quantitative sandwich enzyme immunoassay technique. Serum BCMA (TNFRSF17) was quantitated in serum by ELISA using 96 well plates (MyBioSource, Rochester, NY). This assay also employs the quantitative sandwich enzyme immunoassay technique. Antibody specific for BCMA (TNFRSF17) has been precoated onto a microplate. APRIL was determined in the serum, using a kit from Bender MedSystems (Vienna, Austria). An anti-APRIL polyclonal coating antibody is adsorbed onto microwells.

TNF-*α* was done by using TNF-*α* ELISA kits provided by AviBon, Helsinki, Finland. The TNF-*α* ELISA is an enzyme-linked immunosorbent assay for the quantitative detection of human TNF-*α* in cell culture supernatants and plasma. High sensitive C-reactive protein (hs-CRP) in serum was performed using ELISA technique and interpreted according to the American Heart Association (CRP values < 1.0 mg/L = low risk, CRP values: 1.0–3 mg/L = intermediate risk, and CRP values > 3.0 mg/L = high risk) [12].

### 2.4. Statistical Methods

Statistical package of social science (SPSS) version 15.0 was used for analysis of data. Data was summarized as mean and SD, percentage. *t*-test was used for analysis of two pieces of quantitative data. One way ANOVA test was used for comparison of more than 2 pieces of quantitative data followed by post-HOCC test for detection of significance. Pearson's and Spearman correlation was also done. *r* was consider weak if <0.25, mild if ≥0.25–<0.5, moderate if ≥0.5–<0.75, and strong if ≥0.75. *P* value was considered significant if <0.05^*^.

## 3. Results

### 3.1. Demographic Data of Patients and Controls

Thirty male patients and 20 healthy male controls were recruited for this study. Patients' ages ranged from 16 to 54 years with a mean of 34.3 ± 9.9 years. Their disease duration* ranged from 0.08 to 21 yrs, and median was 1*. For the control group, ages of participants ranged from 23.0 to 50.0 years with mean of 33.1 ± 8.1 years. No significant age difference was found between patients and controls (*P* 0.53).

### 3.2. Results of Clinical Data of Patients

Only two patients (6.7%) of the thirty studied had positive family history and 17 patients (56.7%) had positive pathergy test. The patients' BDCAF index ranged from 1 to 7 with mean of 3.4 ± 1.4. The clinical findings and results of both opthalmological and vascular assessment are represented (Table 1). Twenty patients were on low dose steroids with doses ranging from 5 to 30 mg per day, with or without colchicine and/or azathioprine. The remaining patients were on colchicine and/or azathioprine without steroids.

### 3.3. Results of Laboratory Data

The mean serum levels of TNF-*α*, APRIL, BCMA, and BAFF were higher in patients than in controls in a statistically significant manner (*P* < 0.001) (Table 2). hs-CRP and ESR were significantly elevated in the patient group in relation to control *P* < 0.001 (Table 2).

### 3.4. Correlation between Demographic, Clinical, and Laboratory Data

Positive significant correlation was observed between BDCAF and hs-CRP (*r* 0.68, *P* < 0.001). However no correlation was detected between BDCAF and any of the TNF family members investigated (TNF-*α*, APRIL, BAFF, and BCMA serum levels) (*r* 0.13,* P* 0.5;* r* 0.22,* P* 0.4;* r* 0.19,* P* 0.48;* r* 0.21, and* P* 0.23, resp.).

A significantly higher TNF-*α* level in patients with ocular lesions (*P* 0.007) was observed. There was no statistical difference in serum levels of APRIL, BCMA, or BAFF in patients with or without clinical manifestation. None of the TNF family members tested was affected by a positive pathergy test.

Only serum APRIL was correlated significantly with hsCRP (*r* 0.34, *P* 0.047).

An inverse significant correlation existed between the duration of the disease and the serum levels of APRIL, BCMA, and BAFF (*r* 0.34, *P* 0.048; *r* 0.48, *P* 0.006, *r* 0.42; *P* 0.013, resp.), while with the TNF-*α* the correlation was nonsignificant (*r*  −0.2, *P* 0.3).

### 3.5. Correlation of Different Studied TNF Family Members within Patients Group

The only positive correlations detected were between BCMA, TNF-*α*, and APRIL, where BCMA level correlated positively with both serum TNF-*α* and APRIL levels (*r* 0.71, *P* < 0.001, and *r* 0.34, *P* 0.047, resp.). Also APRIL correlated significantly with TNF-*α* (*r* 0.19, *P* 0.048) (Table 3).

## 4. Discussion

In the present study we detected significantly higher serum levels of TNF-*α*, BAFF, and its homolog APRIL together with their receptor BCMA in patients with Behcet's disease (BD) in comparison to controls. Similarly, other investigators reported an increased TNF-*α* level in BD patients, especially in the exacerbation period [13–15]. Review of the literature revealed one study concerning BAFF in neuro-Behcet, where the cerebrospinal fluid (CSF) levels of BAFF messenger RNA (mRNA) were reported to be upregulated in central nervous system of neuro-Behcet's disease [16]. It has also been demonstrated that BAFF is upregulated in the peripheral circulation and skin biopsies from BD patients [1]. To the best of our knowledge, no previous reports investigated the levels of APRIL and BCMA in Behcet's disease.

Apart from TNF-*α*, the serum levels of BCMA, APRIL, and BAFF correlated negatively with disease duration. Of the TNF family members we examined, TNF-*α* may be the most crucial member for disease maintenance and it seems that TNF-*α* downregulation in response to disease improvement in patients under treatment may be delayed. This implication is supported by the finding that BDCAF of our patients, who were all under treatment, did not correlate significantly with serum levels of TNF-*α*, while Durmazlar et al. [17] reported that TNF-*α* correlates with BDCAF in untreated BD patients. Treatment may suppress inflammation and activity, as measured by BDCAF index and hsCRP, with minimal or delayed effect on TNF-*α*.

The ESR and CRP have been recognized as crude markers of disease activity in BD [18]. Indeed, in the current study, ESR and hs-CRP were more elevated in patients' group than controls in a statistically significant manner. Similar to other investigators, positive correlation between BDCAF and hs-CRP was detected in our study [14, 18, 19]. Interestingly, hs-CRP did not lose its predictive value as a disease activity marker in our patients.

The precise mechanisms of tissue destruction in BD have not been fully elucidated. Nevertheless, a growing number of reports have involved T and B lymphocytes mediated immune responses [20, 21] that may be triggered by bacteria in a yet unknown mechanism. Activated T cells, capable of producing IFN-*γ* and TNF-*α*, are present in the peripheral blood in significantly higher numbers in patients with BD than in those with recurrent aphthous stomatitis (RAS) and in healthy controls [22]. These activated CD4^+^ and CD8^+^ T cells can be involved in the recruitment of neutrophils to the site of inflammation [23]. The neutrophil function is regulated and maintained by several T cell cytokines including IL-1, IL-2, IL-6, IL-8, and TNF-*α*, among others. Neutrophils have enhanced chemotaxis and phagocytosis and induce superoxide generation and myeloperoxidase expression. Neutrophils accumulate in inflammatory lesions where they induce neutrophilic vasculitis [24].

BAFF and its homolog APRIL, which are expressed by neutrophils, may be indirectly involved in regulation of neutrophils, through upregulating IL-6 and GMCSF. BAFF and its homolog APRIL are members of the tumor necrosis factor family and are expressed by monocytes, DCs, neutrophils, basophils, stromal cells, activated T cells, activated and malignant B cells, and epithelial cells [7]. APRIL and/or BAFF trigger nuclear factor-*κ*B activation and IL-6 and granulocyte macrophage colony-stimulating factor (GM-CSF) expression through functional BCMA receptors, an activation inhibited by anti-BCMA short hairpin RNA [25]. This can explain how the levels of both serum GM-CSF and serum IL-6 were found to be high in Behcet's disease as reported by different studies [26, 27].

BCMA stimulation in turn stimulates B cell and, possibly, T cell responses locally. It has been reported that B cell abnormalities could be involved in the pathogenesis of BD where increased levels of activated and memory B cell subsets had been previously demonstrated suggesting a modified B cell function in BD [16]. Increased serum levels of BAFF and its homolog APRIL are found in several autoimmune diseases, and both cytokines can be elaborated in inflammatory sites [28].

TNF-*α* is involved in the blood retinal barrier breakdown by opening tight junctions of retinal vascular endothelial cells and retinal pigmented epithelial (RPE) cells [29]. Our study showed that only TNF-*α* levels in the serum of patients withactive ocular manifestations were significantly higher than inactive ocular BD patients. Our results were in agreement with Santos Lacomba et al. [30] who reported that aqueous humor and sera of patients with uveitis showed significantly higher levels of TNF-*α* than those of healthy controls. On the other hand, we did not find any statistically significant difference between patients with or without ocular manifestations regarding the serum levels of BAFF, APRIL, and BCMA indicating a minor role of these TNF family members in the pathogenesis of ocular manifestations of BD in comparison to TNF-*α*.

None of the TNF family members (TNF-*α*, APRIL, BCMA, and BAFF) we investigated showed any significant difference in expression between patients with or without other active clinical manifestations (oral ulcers, genital ulcers, vascular and cutaneous lesions, and pathergy test). Bozoglu et al. [31] studied 36 patients with BD (divided into 3 groups: patients with acute thrombosis, chronic thrombosis, and mucocutaneous involvement) and found no significant difference regarding TNF-*α* when patient subgroups were compared to each other. However, Durmazlar et al. [17] reported that, in 70 untreated patients with BD, the serum levels of TNF-*α* have strong association with oral ulcer, genital ulcer, the presence of positive pathergy test, and vascular lesion, but no association was found between TNF-*α* levels and erythema nodosum or arthritis. Moreover, Hamzaoui et al. [16] found that BAFF is upregulated in BD and that it correlated with number of skin lesions. This discrepancy may be a result of different study settings including therapeutic intervention and still needs further larger scales studies for verification.

In conclusion, patients with BD have significantly higher inflammatory markers (ESR and hs-CRP) as well as several of the TNF family members' (TNF-*α*, BAFF/APRIL, and BCMA) as compared to controls. Our findings suggest that TNF-*α*, BAFF/APRIL, and BCMA might contribute to the pathogenesis of BD possibly through augmentation of both innate and adaptive immune responses as well as by collaborating with other inflammatory cytokines to promote the activation and differentiation of effecter immune cells involved in the pathogenesis of BD.

The limitation of this study is the small sample size.

## Figures and Tables

**Table 1:** Frequency distribution of clinical data of patients included in the study.

	Number (%)
Family history:	
Negative	28 (93.3)
Positive	2 (6.7)
Ocular examination:	
Negative	18 (60)
Positive	12 (40)
Vascular:	
Inactive	24 (80)
Active	6 (20)
Pathergy test:	
Positive	17 (56.7)
Negative	13 (43.3)
Oral ulcers^#^:	
Absent	11 (36.7)
Present	19 (63.3)
Genital ulcer:	
Absent	16 (53.3)
Present	14 (46.7)
Cutaneous lesion:	
Negative	16 (53.3)
Acneiform	9 (30)
Erythema nodosa	5 (16.7)

^#^Oral ulceration occurred in all patients throughout the course of the disease but some patients had no ulcer at time of examination.

**Table 2:** Laboratory data of patients and controls.

	Cases		Controls		*P* value
	Range	Mean ± standard deviation	Range	Mean ± standard deviation	
ESR (mm/hr)	3–100	30.8 ± 26.5	2–10	6.3 ± 2.56	0.001
hs-CRP (ug/dL)	1.7–30	14.3 ± 10.4	1.11–10.4	4.9 ± 3.15	0.001
TNF-*α* (pg/dL)	2.3–58.9	31 ± 16.8	1.4–13.9	5.08 ± 3.19	0.001
APRIL (ng/mL)	1.64–20.8	8.69 ± 4.9	1.02–6.04	3.13 ± 1.52	0.001
BAFF (Pg/mL)	177–914.9	391.83 ± 145.55	109–398	243.6 ± 81.16	0.001
BCMA (ng/mL)	1.04–27.1	14.1 ± 6.6	1–3.89	2.06 ± 0.71	0.001

ESR: erythrocyte sedimentation rate, hs-CRP: highly sensitive C-reactive protein, TNF-*α*: tumor necrosis factor alpha, BCMA: C cell maturation antigen, APRIL: a proliferation inducing ligand, and BAFF: B cell activation factor.

**Table 3:** Correlations of TNF-*α*, APRIL, BCMA, and BAFF within the patients' group.

	BCMA	APRIL	BAFF
TNF-*α*	*R* 0.94 *P* < 0.001^*^	*R* 0.19 *P* 0.048^*^	*R* 0.25 *P* 0.221
BAFF	*R* 0.219 *P* 0.221	*R* 0.26 *P* 0.261	—
BCMA	—	*R* 0.34 *P* 0.047^*^	*R* 0.219 *P* 0.221

^*^ *P* value is significant <0.05.
